# Calcium hydroxyapatite suppresses extracellular matrix-related gene expression by modulating Piezo1-associated Ca^2+^ signaling and PKA activity

**DOI:** 10.1080/19768354.2026.2677272

**Published:** 2026-08-11

**Authors:** ChanHui Song, Younjee Jeong, Tae-Jin Kim

**Affiliations:** a Department of Integrated Biological Science, College of Natural Sciences, Pusan National University, Busan, Republic of Korea; b Busan Foreign School, Busan, Republic of Korea; c Department of Biological Sciences, College of Natural Sciences, Pusan National University, Busan, Republic of Korea; d Institute of Systems Biology, Pusan National University, Busan, Republic of Korea

**Keywords:** Extracellular matrix, Ca^2+^, Calcium hydroxyapatite, fibrosis, PKA

## Abstract

Fibrosis is characterized by excessive extracellular matrix (ECM) deposition driven by sustained pro-fibrotic signaling and underlies a wide range of pathological conditions. Accordingly, strategies that broadly suppress ECM production are considered promising for the treatment of fibrosis. Although calcium hydroxyapatite (CaHA) is primarily used as a nanoparticle-based dermal filler, previous observations that it affects collagen production suggest its potential repurposing as a regulator of ECM-related programs. Here, we investigated the anti-fibrotic efficacy of CaHA and the signaling mechanisms underlying its effects. CaHA broadly suppressed ECM-related gene expression in fibroblasts, extending beyond collagen to multiple ECM components and associated regulatory factors. Mechanistically, CaHA attenuated Piezo1-associated Ca^2+^ signaling and was also associated with activation of the PKA pathway. Importantly, CaHA similarly reduced ECM-related gene expression in a fibrotic liver cell model, supporting the broader relevance of its anti-fibrotic activity. Together, these findings identify CaHA as a broad suppressor of ECM-related gene expression through modulation of Piezo1-associated Ca^2+^ signaling and PKA-associated pathways, and support its potential as an anti-fibrotic therapeutic strategy.

## GRAPHICAL ABSTRACT

**Figure:**
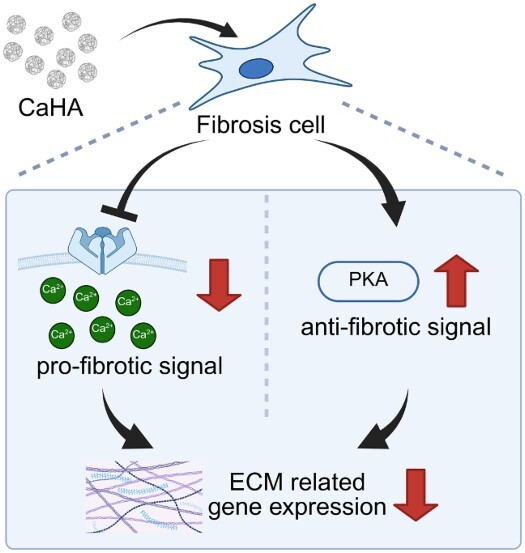

## Introduction

Fibrotic diseases are characterized by excessive deposition and remodeling of the extracellular matrix (ECM), ultimately leading to progressive tissue stiffening, architectural distortion, and loss of organ function across diverse tissues (Rockey et al. 2015). Beyond serving as a structural scaffold, the ECM actively shapes the biochemical and mechanical microenvironment perceived by resident cells. In pathological settings, aberrant ECM remodeling can reinforce pro-fibrotic signaling and establish a self-sustaining feedback loop that drives disease progression (Herrera et al. 2018). Accordingly, therapeutic strategies that directly target ECM accumulation or restore ECM homeostasis have become central to efforts aimed at limiting fibrosis (Wight and Potter-Perigo 2011).

ECM components serve diverse functions, including mechanical support, growth factor sequestration, and regulation of cell adhesion, migration, and differentiation (Kähäri et al. 1991; Corsi et al. 2002; Frantz et al. 2010). Their synthesis and turnover are tightly controlled by multiple intracellular signaling networks (Tada et al. 2001; Weng et al. 2015). Among these, cytosolic Ca^2+^ signaling has emerged as an important integrator that links extracellular cues to ECM-regulating transcriptional programs (Herum et al. 2013; Rahaman et al. 2014). In this context, the mechanosensitive Ca^2+^ channel Piezo1 has attracted attention as a potential mediator coupling mechanical stimuli to ECM-associated cellular responses (Rashidi et al. 2025; Wang et al. 2025). In parallel, other signaling pathways, including cAMP/PKA-associated pathways, have also been implicated in the regulation of fibrotic and anti-fibrotic programs (Wettlaufer et al. 2017; Feng et al. 2019; Wen et al. 2022). Together, these studies suggest that modulation of signaling pathways associated with ECM regulation may provide an effective means of controlling pathological ECM accumulation.

The development of therapeutic strategies aimed at suppressing ECM synthesis requires several considerations. First, effective interventions should enable broad yet controllable regulation of ECM-related gene expression, ideally by targeting upstream signaling nodes that coordinate fibrotic responses (Walraven and Hinz 2018). Indeed, many anti-fibrotic approaches act by modulating such upstream pathways to reduce matrix deposition and slow disease progression (Kutluk Cenik et al. 2013; Kuronuma et al. 2022; Richeldi et al. 2025). Additionally, it is important to determine whether such effects are maintained under pathological microenvironmental conditions, particularly in mechanically active fibrotic settings, where increased matrix stiffness and stress can alter signaling behavior and potentially reduce therapeutic responsiveness (Kalli et al. 2023). These issues are especially relevant in fibroblast-driven fibrosis and in tissues where persistent mechanical stimulation contributes to disease progression.

Calcium hydroxyapatite (CaHA) is a clinically approved biomaterial that has been used widely in aesthetic and reconstructive medicine. In addition to its established clinical applications, CaHA has been reported to influence collagen-related responses (Rakshit et al. 2020), raising the possibility that it may modulate ECM-regulatory programs relevant to fibrotic pathology. However, it remains unclear whether CaHA-associated ECM reduction is limited to selected matrix components or reflects a broader suppression of ECM-related genes. Furthermore, the upstream signaling events associated with this response, and the extent to which the response is preserved under pro-fibrotic conditions, have not been systematically examined.

In the present study, we investigated the extent of ECM-related gene regulation by CaHA in fibroblasts under basal and pro-fibrotic conditions. Using real-time live-cell imaging, we monitored CaHA-associated changes in intracellular Ca^2+^ signaling and examined the contribution of Piezo1-related signaling through pharmacological activation and overexpression approaches. We also evaluated the involvement of PKA-associated signaling in the regulation of ECM-related gene expression. Together, our results identify CaHA as a broad modulator of ECM-associated gene programs and support a model in which CaHA influences anti-fibrotic responses through distinct signaling axes associated with intracellular Ca^2+^ regulation and PKA activity.

## Materials and methods

### Cell culture

Hs68 human dermal fibroblasts were purchased from the American Type Culture Collection (ATCC; CRL-1635), and LX-2 human hepatic stellate cells were kindly provided by Dr. Youngmi Jeong. Both Hs68 and LX-2 cells were cultured in Dulbecco’s Modified Eagle Medium (DMEM; GenDEPOT, CM002-050) supplemented with 10% fetal bovine serum (FBS; Gibco, 16000-044) and 1% penicillin–streptomycin (100 U/mL penicillin and 100 μg/mL streptomycin; GenDEPOT, CA005). Cells were maintained at 37 °C in a humidified incubator with 5% CO₂ and used for experiments at 70–80% confluency.

### DNA constructs and transfection

The D3cpv FRET biosensor for monitoring intracellular Ca^2+^ levels and Piezo1–tdTomato were kindly provided by Dr. Yingxiao Wang (University of California, San Diego). The EKAREV-NLS FRET biosensor used to measure nuclear ERK activity was constructed as previously described (Kim et al. 2022). The hyBRET-PKA-EV biosensor for monitoring PKA activity was provided by Dr. Michiyuki Matsuda. To measure cytoplasmic ROCK activity, the ROCK-NES (mNG) FRET biosensor was generated by replacing the donor fluorophore with mNeonGreen and substituting the membrane-targeting lyn sequence with a nuclear export signal (NES) in the Eevee-ROCK-lyn construct provided by Dr. Michiyuki Matsuda. This modification enabled selective measurement of ROCK activity in the cytoplasm. Cells were seeded to reach approximately 70–80% confluency at the time of transfection. Plasmid DNA was transfected using X-tremeGENE 360™ transfection reagent (Roche, 86925900) according to the manufacturer’s instructions.

### Cell viability assay

Cell viability was assessed using the PrestoBlue™ HS Cell Viability Reagent (Invitrogen). Cells were seeded in 96-well plates and treated as indicated. At the designated time points, the reagent was added directly to the culture medium according to the manufacturer’s protocol and incubated at 37 °C. Fluorescence intensity was measured using a microplate reader (excitation/emission: 560/590 nm).

Cell viability was calculated by subtracting background fluorescence from blank wells (medium plus PrestoBlue™ reagent) and normalizing each condition to the vehicle control. All conditions were tested in at least triplicate wells and independently repeated at least three times.

### Reagents

Calcium hydroxyapatite (CaHA) was purchased from Sigma-Aldrich (677418). For cell treatment, CaHA was diluted to the indicated concentrations following a previously described protocol (Rakshit et al. 2020). Briefly, CaHA was diluted in an aqueous solution prepared by diluting 0.1% NaCl in molecular-grade water. GsMTx4 was obtained from Alomone Labs (STG-100), and forskolin was purchased from MedChemExpress (HY-15371). Yoda1 was purchased from Sigma-Aldrich (SML1558) and dissolved in dimethyl sulfoxide (DMSO; Biosesang, AC4002-050-00). DMSO was used as a vehicle control at a final concentration of 0.5% (v/v). For each experiment, control groups were treated with the corresponding dilution solution used for the experimental condition.

### FRET live imaging

Live-cell fluorescence resonance energy transfer (FRET) imaging was performed using cells seeded in glass-bottom confocal dishes (SPL, 200350) and transfected with the indicated FRET biosensors. Imaging was conducted on a Leica DMi8 inverted fluorescence microscope equipped with an LED8 light source, an HC PL APO 100×/1.30 oil immersion objective, a DIC module, and a K5 sCMOS camera (Leica Microsystems). A stage-top incubation system was used to maintain physiological temperature and CO₂ levels throughout image acquisition. For FRET measurements, cells were excited at 440 nm, and emission signals were collected in the cyan fluorescent protein (CFP) and FRET channels using a CYR71010 filter cube (Leica, 11525416) mounted on a motorized emission filter wheel (Leica, EFW-LED8). Image acquisition was performed using LAS X software (Leica Microsystems). Exposure time and detector gain were kept constant across conditions within each experiment. Fluorescence images were acquired at 1-min intervals for time-lapse ratiometric analysis. All live-cell imaging experiments were independently repeated at least three times under matched experimental conditions.

### FRET ratio analysis

For quantitative analysis of FRET signals, the subcellular region in which the biosensor was localized was selected as the region of interest (ROI). For each image, background fluorescence was measured in a cell-free area and subtracted from the ROI signal in both the FRET and CFP channels to correct for nonspecific fluorescence and camera noise. The background-corrected FRET/CFP ratio was calculated on a pixel-by-pixel basis according to the following equation:

$$\mathrm{FRET}/\mathrm{CFP}\;\mathrm{ratio}=\;\frac{\left[I(\mathrm{FRE}T_{\mathrm{ROI}})-I(\mathrm{FRE}T_{\mathrm{bg}})\right]}{\left[I(\mathrm{ECF}P_{\mathrm{ROI}})-I(\mathrm{ECF}P_{\mathrm{bg}})\right]}$$

where *I* denotes the fluorescence intensity measured in the indicated channel and region. The resulting FRET ratio images were visualized using an intensity-modified display mode, in which the color of each pixel represents the local FRET/CFP intensity ratio. In the FRET biosensors used in this study, a CFP (cyan fluorescent protein)-based protein served as the donor, whereas a YFP (yellow fluorescent protein)- or GFP (green fluorescent protein)-based protein served as the acceptor for activity measurement.

### Quantitative real-time PCR (qPCR)

Total RNA was isolated from cells using an RNA extraction kit according to the manufacturer’s instructions. Purified RNA was quantified by spectrophotometry and reverse-transcribed into complementary DNA (cDNA) using a commercial reverse transcription kit. Quantitative PCR was performed on a real-time PCR system using a SYBR Green–based master mix. Reactions were prepared with cDNA template, SYBR Green master mix, and gene-specific primers (Table 1), and run under standard cycling conditions consisting of an initial denaturation step followed by 45 cycles of denaturation and annealing. Melt-curve analysis was performed to confirm amplification specificity. GAPDH was used as the internal reference gene for normalization. Relative gene expression levels were calculated using the 2^(ΔΔCt) method. Each sample was analyzed in technical triplicate, and all experiments were independently repeated at least three times.

**Table 1.** Primer list used in qPCR experiments.

Gene name	Forward	Reverse
COL1A1	GATTCCCTGGACCTAAAGGTGC	AGCCTCTCCATCTTTGCCAGCA
COL4A1	GGCAGATTCGGACCACTAGG	GCGTCTGTGGCAATACTAGC
COL5A1	CTTGGCCCAAAGAAAACCCG	GTAGGTGACGTTCTGGTGGG
COL6A3	CCTGGTGTAACTGATGCTGCCA	AAGATGGCGTCCACCTTGGACT
COL11A1	CAATAGCACAGACGGAGGCA	CCCAGAAACATGCCTAGGAGC
FN1	CTGGCCGAAAATACATTGTAAA	CCACAGTCGGGTCAGGAG
BGN	TTGAACCTGGAGCCTTCGATGG	TTGGAGTAGCGAAGCAGGTCCT
FMOD	CAGTCAACACCAACCTGGAGAAC	CAGCTTGGAGAAGTTCACGACG
CTGF	GCAGGCTAGAGAAGCAGAGC	ATGTCTTCATGCTGGTGCAG
Piezo1	CATCATCCCCTTCACGGCCC	CCGGAGCTGCCCTCAATCTG
GAPDH	CGACCACTTTGTCAAGCTCA	AGGGGTCTACATGGCAACTG

### Statistical analysis

All data are presented as mean ± standard error of the mean (SEM). Statistical analyses were performed using GraphPad Prism version 10.1.2. Data normality was assessed prior to parametric testing. Differences between two groups were evaluated using unpaired, two-tailed Student’s t-tests. A *p*-value < 0.05 was considered statistically significant. Statistical significance is indicated in the figures as follows: *(*p* < 0.05), **(*p* < 0.01), ***(*p* < 0.001), and ****(*p* < 0.0001). Unless otherwise stated, all experiments were performed with at least three independent biological replicates.

## Results

### CaHA suppresses broad ECM-related gene expression in fibroblasts

**Figure 1.**
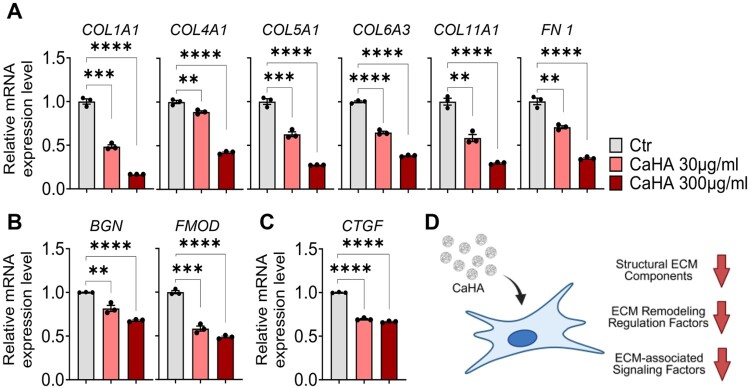
CaHA-associated changes in the mRNA expression of ECM-related genes in fibroblasts. (A-C) Relative mRNA expression levels in Hs68 cells treated for 48 h with vehicle control or CaHA (30 or 300 μg/mL). Gene expression was quantified for ECM structural components (A), ECM regulatory factors (B), and ECM signaling factors (C) (n = 3). (D) Schematic summary of the results showing CaHA-induced transcriptional changes in Hs68 cells.

To define the extent of CaHA-mediated suppression of ECM-related genes, we used Hs68 human dermal fibroblasts as a normal fibroblast model. After establishing CaHA treatment conditions suitable for expression analysis (Supplementary Figure S1A), we quantified CaHA-induced changes in ECM-related gene expression (Figure 1(A–C)). The qPCR panel included genes encoding major ECM components (Lee et al. 2024), as well as regulators of ECM organization and remodeling, such as BGN and FMOD, which are proteoglycans known to contribute to matrix assembly and homeostasis (Genovese et al. 2013; Zheng et al. 2023). We also included CTGF, a mechanosensitive pro-fibrotic mediator closely associated with ECM production and tissue remodeling (Shi-Wen et al. 2008). At 48 h after treatment, the previously reported suppression of COL1A1 expression was reproducibly observed under identical conditions (Rakshit et al. 2020), confirming the robustness of our experimental setup (Figure 1(A)). Notably, the inhibitory effect of CaHA was not limited to COL1A1 but extended to multiple collagen subtypes as well as fibronectin (Figure 1(A)). In addition, CaHA treatment significantly altered the expression of genes involved in ECM remodeling (Figure 1(B)) and mechanotransduction-related pathways (Figure 1(C)). Collectively, these results demonstrate that CaHA broadly suppresses the transcription of ECM components and associated regulatory factors in fibroblasts (Figure 1(D)).

### Sustained CaHA treatment suppresses cytosolic Ca^2+^ levels

**Figure 2.**
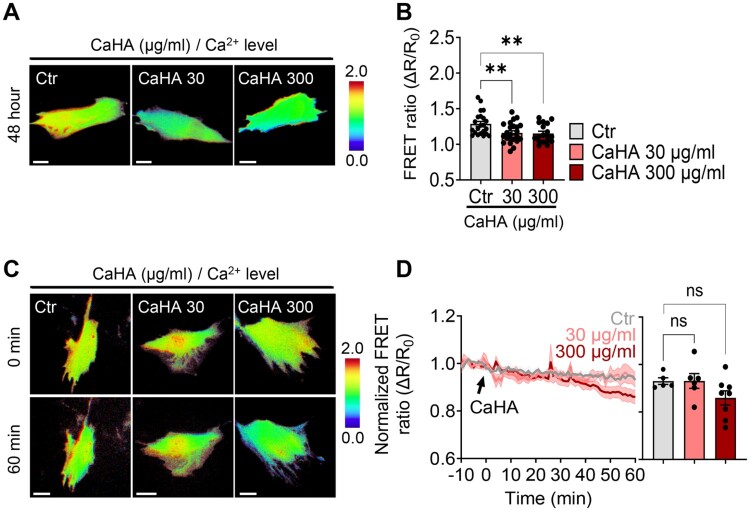
CaHA-associated changes in intracellular Ca^2+^ levels in fibroblasts. (A,B) Representative pseudocolor FRET/CFP ratio images of Hs68 cells expressing the D3cpv Ca^2+^ biosensor after 48 h treatment with vehicle control or CaHA (30 or 300 μg/mL) (A). Quantification of the D3cpv FRET/CFP ratio at 48 h is presented as a bar graph (B) (n = 18–23). (C,D) Representative D3cpv FRET/CFP ratio images acquired immediately before treatment (0 min) and after 1 h of acute exposure to vehicle control or CaHA (30 or 300 μg/mL) (C). Time-lapse quantification of the FRET/CFP ratio was performed at 1-min intervals, from 5 min prior to treatment to 60 min post-treatment, and the bar graph represents the endpoint (60 min) ratio (D) (n = 5–8). Scale bars, 20 μm.

Following the observation that CaHA suppresses ECM-related gene expression in fibroblasts, we next examined whether CaHA affects upstream signaling pathways associated with ECM-related gene expression. Cytoplasmic Ca^2+^ influx is a key signaling event implicated in the regulation of ECM production (Herum et al. 2013; Rahaman et al. 2014; Kim et al. 2024). To assess the impact of CaHA on Ca^2+^ signaling, intracellular Ca^2+^ levels were monitored using the validated D3cpv FRET biosensor (Palmer et al. 2006). We first analyzed changes in cytosolic Ca^2+^ levels during prolonged CaHA exposure using live-cell imaging over a 48 h period (Figure 2(A)). Under these conditions, cytosolic Ca^2+^ levels were significantly reduced compared with those in control cells at 48 h after CaHA treatment (Figure 2(B)). To determine whether CaHA also induces acute changes in Ca^2+^ signaling, we performed short-term real-time imaging for 1 h using the same biosensor (Figure 2(C)). In contrast to the long-term treatment, no statistically significant change in cytosolic Ca^2+^ levels was observed during this acute time window (Figure 2(D)). Together, these results indicate that CaHA suppresses cytosolic Ca^2+^ levels in a time-dependent manner, with significant effects emerging upon sustained treatment.

### CaHA suppresses Piezo1-associated ECM-related gene expression

**Figure 3.**
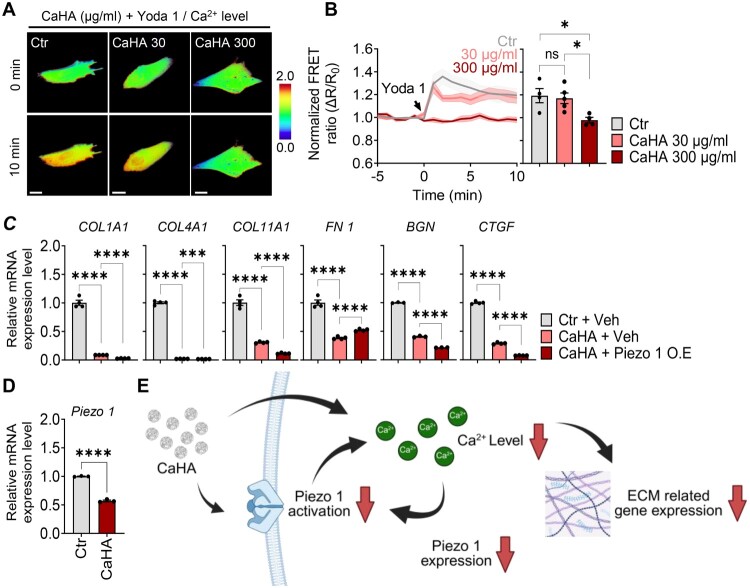
Piezo1-associated changes in Ca^2+^ dynamics and ECM-related gene expression following CaHA treatment. (A,B) Representative pseudocolor FRET/CFP ratio images of Hs68 cells expressing the D3cpv Ca^2+^ biosensor stimulated with Yoda1 (1 μM) after 48 h treatment with vehicle control or CaHA (30 or 300 μg/mL) (A). Time-lapse quantification of the FRET/CFP ratio was performed at 1-min intervals, from 5 min prior to Yoda1 stimulation to 10 min post-stimulation, and the bar graph represents the endpoint (10 min) ratio (B) (n = 4–5). Scale bars, 20 μm. (C) Relative mRNA expression levels of ECM components in Hs68 cells treated with vehicle control or CaHA (300 μg/mL) for 48 h, comparing conditions with or without Piezo1 overexpression (n = 3–4). (D) Relative Piezo1 mRNA expression levels in Hs68 cells treated with vehicle control or CaHA (300 μg/mL) for 48 h (n = 3). (E) Schematic diagram illustrating the proposed mechanism by which CaHA suppresses Piezo1-associated Ca^2+^ signaling, leading to reduced ECM-related gene expression and decreased Piezo1 expression.

To assess whether CaHA-induced changes in cytosolic Ca^2+^ signaling are associated with the mechanosensitive Ca^2+^ channel Piezo1 (Rashidi et al. 2025; Wang et al. 2025), we examined the effects of CaHA under conditions of Piezo1 hyperactivation induced by the Piezo1 agonist Yoda1 (Botello-Smith et al. 2019) (Figure 3(A,B)). Following 48 h of CaHA treatment, the Yoda1-induced increase in cytosolic Ca^2+^ levels was significantly attenuated (Figure 3(B)), and this inhibitory effect was reduced in a CaHA concentration–dependent manner. We next evaluated whether CaHA affects ECM-related gene expression under conditions of Piezo1 overexpression (Figure 3(C)). Under CaHA-treated conditions, Piezo1 overexpression did not fully restore the expression of ECM-related genes, although the response pattern differed from that of control cells. This difference may reflect the distinction between increased channel abundance and direct channel activation, as overexpression alone does not necessarily lead to a proportional increase in Piezo1 activity or downstream Ca^2+^-dependent transcriptional responses, whereas Yoda1 directly promotes channel gating and induces a more robust acute Ca^2+^ influx.

Given previous reports that Piezo1 expression itself can be regulated by Ca^2+^ signaling (Peng et al. 2025), we further examined whether CaHA alters Piezo1 expression levels (Figure 3(D)). Consistent with this possibility, Piezo1 expression was significantly reduced following CaHA treatment. These results suggest that CaHA suppresses Piezo1-associated Ca^2+^ signaling and modulates the expression of a subset of ECM-related genes. However, because Piezo1 overexpression did not fully rescue the CaHA-induced gene expression changes, the present data support an important association of the Piezo1-Ca^2+^ axis with the anti-fibrotic effects of CaHA, rather than indicating that Piezo1 alone fully accounts for this response (Figure 3(E)).

### CaHA suppresses ECM-related gene expression in association with PKA activation

**Figure 4.**
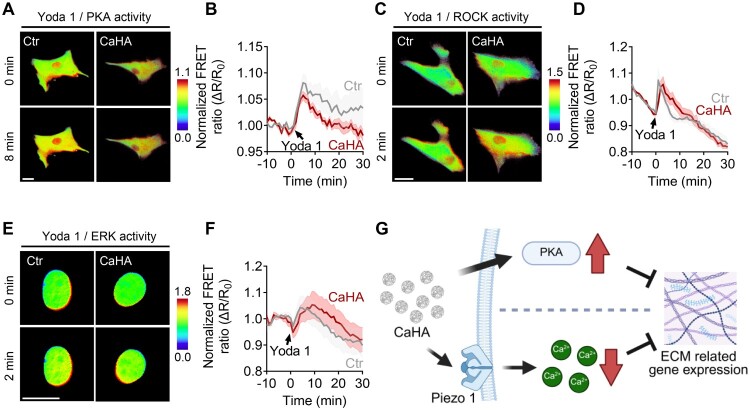
CaHA-associated changes in signaling pathways related to ECM regulation following Yoda1 stimulation. (A–F) Representative pseudocolor FRET/CFP ratio images of Hs68 cells expressing the indicated biosensors following 48 h treatment with vehicle control or CaHA (300 μg/mL) and subsequent stimulation with Yoda1 (1 μM): hyBRET-PKA-EV (A), ROCK-NES (mNG) (C), and EKAREV-NLS (E). (B,D,F) Time-lapse quantification of the FRET/CFP ratio was performed at 1-min intervals from 10 min prior to Yoda1 stimulation to 30 min post-stimulation, for hyBRET-PKA-EV (B), ROCK-NES (mNG) (D), and EKAREV-NLS (F) (n = 4–6). Scale bars, 20 μm. (G) Schematic diagram illustrating the effects of CaHA on cell signaling pathways associated with reduced ECM-related gene expression.

We next asked whether CaHA regulates ECM-related gene expression through signaling pathways other than Piezo1-associated Ca^2+^ signaling. To address this, we examined the effects of CaHA on PKA, ROCK, and ERK signaling, all of which have been implicated in fibrosis-related cellular responses (Wettlaufer et al. 2017; Feng et al. 2019; Wen et al. 2022). Using FRET-based biosensors, we compared the responsiveness of these pathways under CaHA-treated conditions and, based on previous reports suggesting functional links between Piezo1 and these signaling networks (Kim et al. 2022), we also assessed their responses to Yoda1, a Piezo1 activator. We first monitored PKA activity using the hyBRET-PKA-EV biosensor (Figure 4(A,B)). After 48 h of CaHA treatment, Yoda1 failed to further enhance PKA activity compared with the control group (Figure 4(B)). To determine whether this reduced responsiveness reflected an already elevated basal state, we analyzed the raw imaging data prior to quantification (Supplementary Figure S2A). This analysis showed that CaHA itself increased basal PKA activity. In contrast, CaHA treatment did not suppress Yoda1-induced ROCK activation, as measured using the ROCK-NES (mNG) FRET biosensor (Figure 4(C,D)). Similarly, ERK signaling, assessed using the EKAREV-NLS FRET biosensor, did not show the same inhibitory pattern under CaHA-treated conditions (Figure 4(E,F)).

Because CaHA increased basal PKA activity, we next examined whether pharmacological PKA activation could influence ECM-related gene expression (Supplementary Figure S3A). To this end, cells were treated with forskolin, a well-established activator of the cAMP-PKA pathway (Chen et al. 1998). Forskolin treatment generally reduced the expression of ECM-related genes in Hs68 cells and attenuated the additional suppressive effect of CaHA, consistent with the idea that PKA activation contributes to the transcriptional response induced by CaHA. Together, these results suggest that PKA activation is associated with the CaHA-mediated suppression of ECM-related gene expression. Importantly, this effect is interpreted as a pathway parallel to, rather than linearly downstream of, CaHA-induced regulation of Piezo1-associated Ca^2+^ signaling (Figure 4(G)).

### CaHA maintains its ability to suppress ECM-related gene expression in a fibrotic cellular environment

**Figure 5.**
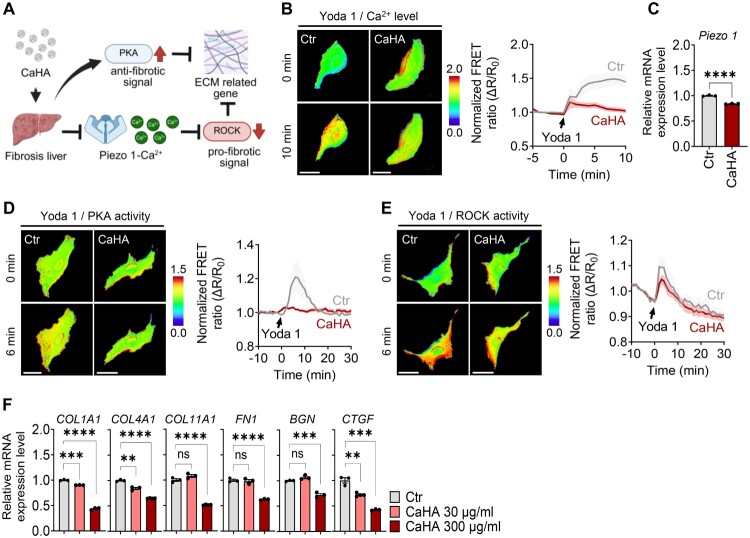
CaHA-associated suppression of ECM-related gene expression and modulation of Piezo1-associated signaling in liver fibrotic cells. (A) Schematic overview of the proposed model in which CaHA suppresses ECM-related gene expression in liver fibrotic cells through modulation of Piezo1-associated Ca^2+^ signaling and PKA activity. (B) Representative pseudocolor FRET/CFP ratio images of LX-2 cells expressing the D3cpv Ca^2+^ biosensor stimulated with Yoda1 (1 μM) after 48 h treatment with vehicle control or CaHA (300 μg/mL). Time-lapse quantification of the FRET/CFP ratio was performed at 1-min intervals, from 5 min prior to Yoda1 stimulation to 10 min post-stimulation (n = 8–9). (C) Relative Piezo1 mRNA expression levels in LX-2 cells treated for 48 h with vehicle control or CaHA (300 μg/mL) (n = 3). (D,E) Representative pseudocolor FRET/CFP ratio images of LX-2 cells expressing hyBRET-PKA-EV (D) or ROCK-NES (mNG) (E), stimulated with Yoda1 (1 μM) following 48 h treatment with vehicle control or CaHA (300 μg/mL). Time-lapse quantification of the FRET/CFP ratio was performed at 1-min intervals, from 10 min prior to Yoda1 stimulation to 30 min post-stimulation (n = 6–11). Scale bars, 50 μm. (F) Relative mRNA expression levels of ECM-related genes in LX-2 cells treated for 48 h with vehicle control or CaHA (30 or 300 μg/mL) (n = 3–4).

Finally, we investigated whether CaHA retains its ECM-suppressive capacity under hyperfibrotic conditions (Figure 5(A)). LX-2 cells were used as an in vitro liver fibrosis model, as they exhibit activated stellate cell–like features and produce fibrotic ECM (Xu et al. 2005). After establishing experimental conditions identical to those used in fibroblast experiments (Supplementary Figure S1B), all subsequent assays were performed under the same conditions. Consistent with the fibroblast data, CaHA pretreatment attenuated Yoda1-evoked increases in cytosolic Ca^2+^ levels (Figure 5(B)) and significantly reduced Piezo1 expression in LX-2 cells (Figure 5(C)). Time-course analysis revealed that CaHA-mediated modulation of cytosolic Ca^2+^ signaling emerged only after prolonged treatment and was not detectable at early time points (Supplementary Figure S4).

We next examined upstream signaling pathways associated with ECM regulation in LX-2 cells using FRET-based biosensors (Figure 5(D,E); Supplementary Figure S2). CaHA increased basal PKA activity in LX-2 cells (Figure 5(D); Supplementary Figure S2B). In contrast, unlike the results observed in dermal fibroblasts, CaHA did not increase basal ROCK activity, but instead attenuated the responsiveness of ROCK signaling to Yoda1 stimulation (Figure 5(E); Supplementary Figure S2C). ERK activity was not detectably altered under these conditions (Supplementary Figure S5). In addition, the effects of pharmacological PKA activation on CaHA responsiveness were consistent with those observed in dermal fibroblasts (Supplementary Figure S3B).

Finally, CaHA treatment dose-dependently suppressed the expression of multiple ECM-related genes in LX-2 cells (Figure 5(F)). Moreover, Piezo1 overexpression did not fully restore ECM-related gene expression following CaHA treatment, a pattern similar to that observed in dermal fibroblasts (Supplementary Figure S6). To further evaluate the involvement of Piezo1-associated signaling, we pharmacologically inhibited Piezo1 using GsMTx4 (Bae et al. 2011). Under these conditions, ECM-related gene expression was reduced, and the additional suppressive effect of CaHA was blunted (Supplementary Figure S7A). Together, these results indicate that CaHA broadly suppresses ECM-related gene expression even in a fibrotic cellular environment, and that this response is accompanied by modulation of Piezo1-associated Ca^2+^ signaling and PKA activity, with an overall pattern that is largely consistent with that observed in dermal fibroblasts.

## Discussion

In this study, we investigated how calcium hydroxyapatite (CaHA) suppresses extracellular matrix (ECM)-related gene expression and assessed its potential relevance as an anti-fibrotic strategy. Although previous work showed that CaHA reduces collagen I expression (Rakshit et al. 2020), its effects on broader ECM-related gene programs and the associated upstream signaling mechanisms had remained unclear. CaHA is of particular translational interest because it can be administered locally, thereby reducing concerns associated with systemic exposure (Ridenour and Kontis 2009). In addition, CaHA has been explored as a drug delivery platform, raising the possibility of combination approaches that may further enhance therapeutic efficacy (Matsumoto et al. 2004; Tomoda et al. 2010). These considerations motivated us to examine the breadth of CaHA-mediated ECM suppression, its signaling basis, and whether these effects are maintained in a fibrotic cellular environment.

Our transcriptional analyses showed that CaHA suppresses a broad spectrum of ECM-related genes in dermal fibroblasts. Consistent with previous reports, COL1A1 expression was reduced, but this effect also extended to multiple collagen subtypes, fibronectin, and genes involved in ECM organization and remodeling (Kähäri et al. 1991; Hedlund et al. 1994; Corsi et al. 2002). In addition, CaHA reduced the expression of ECM-responsive genes linked to mechanotransduction and matrix-dependent signaling (Weston et al. 2003; Dupont et al. 2011; Guo et al. 2011). Together, these findings indicate that CaHA does not act only on individual ECM components, but more broadly modulates transcriptional programs associated with ECM production and remodeling.

To identify upstream signaling pathways involved in these effects, we focused on Ca^2+^ signaling, a central regulator of ECM synthesis and remodeling (Herum et al. 2013; Rahaman et al. 2014). Because Ca^2+^ signaling is highly dynamic, we used FRET-based live-cell biosensors to monitor signaling responses in real time with high temporal resolution (Berridge et al. 2003; DiPilato and Zhang 2010; Choi et al. 2025). Using this approach, we found that sustained CaHA treatment attenuated cytosolic Ca^2+^ signaling. We further observed that CaHA reduced both Piezo1-associated Ca^2+^ responses and Piezo1 expression, consistent with emerging evidence that Piezo1 participates in feedback regulation within Ca^2+^ signaling networks (Peng et al. 2025). At the same time, Piezo1 overexpression did not fully restore ECM-related gene expression under CaHA-treated conditions, indicating that modulation of the Piezo1-associated Ca^2+^ axis alone does not fully explain the transcriptional effects of CaHA.

We therefore examined additional signaling pathways involved in ECM regulation. Among the pathways tested, CaHA was associated with increased basal PKA activity in both dermal fibroblasts and LX-2 cells. Moreover, pharmacological activation of PKA by forskolin recapitulated the suppression of ECM-related gene expression, supporting the view that PKA contributes to the transcriptional response induced by CaHA (Wettlaufer et al. 2017). Importantly, our data do not support a model in which CaHA-induced Ca^2+^ regulation and PKA activation form a single linear signaling cascade. Rather, the current results are more consistent with a model in which modulation of Piezo1-associated Ca^2+^ signaling and activation of PKA represent parallel responses that together contribute to ECM suppression. In contrast to PKA, ROCK was more context dependent. In dermal fibroblasts, CaHA did not suppress Yoda1-induced ROCK or ERK activation, whereas in LX-2 cells CaHA attenuated ROCK responsiveness without detectably altering ERK activity. This pattern suggests that CaHA engages both shared and cell type-dependent signaling responses while converging on a common anti-fibrotic transcriptional outcome.

Several limitations of this study should be acknowledged. First, the present conclusions are based primarily on transcript-level analyses, and representative ECM proteins were not directly evaluated. Future studies will be needed to determine whether the observed suppression of ECM-related gene expression is accompanied by corresponding reductions in ECM protein production and deposition. Second, although our data support roles for Piezo1-associated Ca^2+^ signaling and PKA activation, other major fibrogenic pathways, including the transforming growth factor-β (TGF-β)/SMAD axis, were not directly examined in this study (Meng et al. 2016). Third, while we evaluated both normal dermal fibroblasts and activated hepatic stellate cells, we did not validate the effects of CaHA in an activated fibrotic dermal fibroblast model, such as under TGF-β stimulation or mechanically induced pro-fibrotic conditions. Incorporating these disease-relevant settings, as well as in vivo fibrosis models, will be important in future studies.

In summary, our study identifies CaHA as a broad suppressor of ECM-related gene expression and shows that this effect is associated with modulation of Piezo1-associated Ca^2+^ signaling and activation of PKA. Notably, the ECM-suppressive activity of CaHA is maintained in a fibrotic cellular environment, supporting its potential relevance as an anti-fibrotic intervention. More broadly, these findings suggest that coordinated regulation of mechanosensitive Ca^2+^ signaling and PKA-associated pathways may represent a useful strategy for attenuating fibrotic gene programs while preserving overall cellular signaling balance.

## Supplementary Material

Supplementary Material_TACS-2026-0026-Final.docx
