# Supplementary material for: Calcium hydroxyapatite suppresses extracellular matrix-related gene expression by modulating Piezo1-associated Ca2+ signaling and PKA activity
Source: Anim Cells Syst (Seoul). 2026 Aug 11;30(1):459–70. doi: 10.1080/19768354.2026.2677272 (PMC13463449; doi:10.1080/19768354.2026.2677272)
Supplement: Supplementary file 1 [file TACS_A_2677272_SM6209.docx]

**Supplementary Material**

**Calcium hydroxyapatite suppresses extracellular matrix-related gene expression by modulating Piezo1-associated Ca²⁺ signaling and PKA activity**

ChanHui Song^a^, Younjee Jeong^b^ and Tae-Jin Kim^a,c,d,*^

^a^Department of Integrated Biological Science, College of Natural Sciences, Pusan National University, Busan, Republic of Korea; ^b^Busan Foreign School, Busan, Republic of Korea; ^c^Department of Biological Sciences, College of Natural Sciences, Pusan National University, Busan, Republic of Korea; ^d^Institute of Systems Biology, Pusan National University, Busan, Republic of Korea

**Supplementary Figure S1. Effects of CaHA treatment on cell viability across different concentrations in Hs68 and LX-2 cells.** (**A**) Cell viability of Hs68 cells measured 48 h after treatment with vehicle control or increasing concentrations of CaHA (0.03–3000 μg/mL) (n = 4). (**B**) Cell viability of LX-2 cells measured 48 h after treatment with vehicle control or increasing concentrations of CaHA (0.03–3000 μg/mL) (n = 3).

**Supplementary Figure S2. Quantification of changes in cell signaling activity in Hs68 and LX-2 cells following Yoda1 treatment using raw imaging data.** (A–C) Time-lapse FRET/CFP ratio traces were obtained at 1-min intervals from 10 min prior to Yoda1 stimulation to 30 min after stimulation in hyBRET-PKA-EV-expressing Hs68 cells (A), hyBRET-PKA-EV-expressing LX-2 cells (B), and ROCK-NES (mNG)-expressing LX-2 cells (C) (n = 5–11). Each graph shows the raw time-course data corresponding to the quantified datasets presented in Figure 4B (A), Figure 5D (B), and Figure 5E (C).

**Supplementary Figure S3. Changes in ECM-related gene expression following CaHA treatment in the presence of Forskolin.** (A, B) Relative mRNA expression levels of ECM-related genes in Hs68 (A) and LX-2 (B) cells treated for 48 h with vehicle control, Forskolin (10 μM), or Forskolin (10 μM) plus CaHA (300 μg/mL) (n = 3–4).

**Supplementary Figure S4. Real-time monitoring of cytosolic Ca²⁺ dynamics during acute CaHA exposure in liver fibrotic cells.** (A, B) Representative pseudocolor FRET/CFP ratio images of LX-2 cells expressing the cytosolic Ca²⁺ biosensor D3cpv, acquired immediately before treatment (0 min) and 1 h after acute exposure to vehicle control or CaHA (300 μg/mL) (A). Time-lapse quantification of the FRET/CFP ratio was performed at 1-min intervals from 5 min prior to treatment to 60 min after treatment. The bar graph represents the endpoint FRET/CFP ratio at 60 min (B) (n = 5–6). Scale bars, 20 μm.

**Supplementary Figure S5. CaHA-associated modulation of Yoda1-induced nuclear ERK activity dynamics in liver fibrotic cells.** (A, B) Representative pseudocolor FRET/CFP ratio images of LX-2 cells expressing the nuclear ERK activity biosensor EKAREV-NLS after 48 h of treatment with vehicle control or CaHA (300 μg/mL), followed by stimulation with Yoda1 (1 μM) (A). Time-lapse quantification of the FRET/CFP ratio was performed at 1-min intervals from 10 min prior to Yoda1 stimulation to 30 min after stimulation (B) (n = 4–5). Scale bars, 20 μm.

**Supplementary Figure S6. CaHA-associated changes in ECM-related gene expression under Piezo1 overexpression in liver fibrotic cells.** Relative mRNA expression levels of ECM-related genes in LX-2 cells treated for 48 h with vehicle control or CaHA (300 μg/mL), under conditions with or without Piezo1 overexpression (n = 3).

**Supplementary Figure S7. CaHA-associated changes in ECM-related gene expression under GsMTx4 treatment in liver fibrotic cells.** Relative mRNA expression levels of ECM-related genes in LX-2 cells treated for 48 h with vehicle control, GsMTx4 (2.5 μM), or GsMTx4 (2.5 μM) plus CaHA (300 μg/mL) (n = 3–4).
